# The Identification of Alzheimer’s Disease Using Functional Connectivity Between Activity Voxels in Resting-State fMRI Data

**DOI:** 10.1109/JTEHM.2020.2985022

**Published:** 2020-04-03

**Authors:** Yuhu Shi, Weiming Zeng, Jin Deng, Weifang Nie, Yifei Zhang

**Affiliations:** Information Engineering CollegeShanghai Maritime University 12477 Shanghai201306China

**Keywords:** Activity voxels, fMRI, functional connectivity, independent component analysis, support vector machine

## Abstract

Background: Alzheimer’s disease (AD) is a common neurodegenerative disease occurring in the elderly population. The effective and accurate classification of AD symptoms by using functional magnetic resonance imaging (fMRI) has a great significance for the clinical diagnosis and prediction of AD patients. Methods: Therefore, this paper proposes a new method for identifying AD patients from healthy subjects by using functional connectivities (FCs) between the activity voxels in the brain based on fMRI data analysis. Firstly, independent component analysis is used to detect the activity voxels in the fMRI signals of AD patients and healthy subjects; Secondly, the FCs between the common activity voxels of the two groups are calculated, and then the FCs with significant differences are further identified by statistical analysis between them; Finally, the classification of AD patients from healthy subjects is realized by using FCs with significant differences as the feature samples in support vector machine. Results: The results show that the proposed identification method can obtain higher classification accuracy, and the FCs between activity voxels within prefrontal lobe as well as those between prefrontal and parietal lobes play an important role in the prediction of AD patients. Furthermore, we also find that more brain regions and much more voxels in some regions are activity in AD group compared with health control group. Conclusion: It has a great potential value for the AD pathogenesis mechanism study.

## Graphical abstract

$\boldsymbol { X } _ { i } ( i

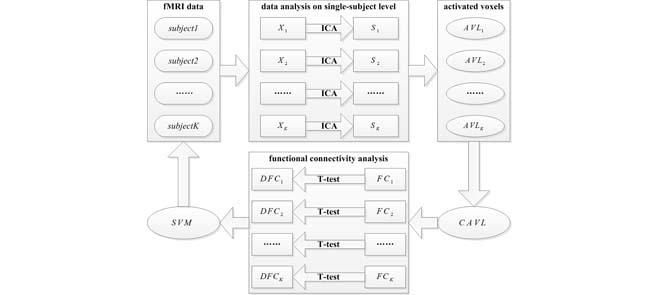

## I. Introduction

Alzheimer’s disease (AD) is a progressive neurodegenerative disease with the characters of memory loss, poor judgment, language deterioration and so on, which is one of the highest rates of disease among the elderly [1]. According to the report released by the AD International (ADI) in 2018, the current cost of the disease is about a trillion US dollars per year, and the number of people worldwide suffer from AD will increase from the current 50 million to 152 million by 2050 [2]. Therefore, the prediction and diagnosis of AD are very important at its early warning, which has become a crucial step to retard or even avoid dementia.

Many neuroimaging techniques have been used to unveil neuroanatomical and neurophysiological substrates of AD so far [3], [4], such as positron emission tomography (PET) [5], diffusion tensor imaging (DTI) [6], electroencephalogram (EEG) [7] and functional magnetic resonance imaging (fMRI) [8]. Thereinto, fMRI is one of the most promising non-invasive techniques for early AD diagnosis [9], [10]. By using fMRI technique, some previous AD studies have demonstrated that the disrupted functional connectivities (FCs) mainly concentrated on several key brain regions of neuronal degeneration in AD, such as the anterior hippocampus [11], inferior parietal lobe [12], thalamus [13], posterior cingulate cortex [14], and prefrontal cortex [15]. These findings suggested that the FCs with significant differences in AD patients compared with healthy subjects can be served as a biomarker for the diagnosis of AD.

In addition, some other studies have tried to study AD using the measures in graph theory, for example, Binnewijzend *et al.* used voxel-wise eigenvector centrality to analyze individual whole-brain resting-state fMRI data and identified changes in brain network organization in AD patients that are related to cognition and underlying AD pathology by a permutation-based method [16]. Many methods can be used to study FCs such as elastic net regularized regression [17] and independent component analysis (ICA) [18]. Among them, ICA is a very popular blind source separation technique that has been fruitfully applied in the AD research, for example, Zhong *et al.* investigated the differences of effective connectivity of the default mode network (DMN) in AD patients and normal controls based on the identification of DMN using ICA [19].

As a simple and effective means of research, classification of healthy and unhealthy individuals for a given disease is a common practice [20]–[24]. Without exception, classification is particularly challenging in developing biomarkers for studies of AD [25]–[27]. Currently, the literatures regarding AD on automatic classification systems based on fMRI are depth and breadth [28], [29]. For example, in order to assists in the diagnosis of AD and supports the monitoring of the progression of the disease, Tripoliti *et al.* proposed a six stage method based on the features extracted from the fMRI data to classify AD patients [30]. In addition, Dai *et al.* proposed a methodological framework called multi-modal imaging and multi-level characteristics with multi-classifier (M3), to discriminate AD patients from healthy controls [31].

Many methods can be used for the classification of AD. Among them, machine learning (ML) and pattern classification techniques have played an important role in exploring the brain differences between AD patients and healthy controls [32]. As the state-of-the-art ML techniques, support vector machine (SVM) and deep learning (DL) has been widely used in brain imaging classification of AD [33], [34]. For example, Khazaee *et al.* combined graph theoretical approaches with SVM to classify AD patients from healthy subjects by studying functional brain network alteration [35]. Sarraf *et al.* outlined state-of-the-art DL-based pipelines employed to distinguish AD’s MRI and fMRI data from normal healthy control data for the same age group, where the fMRI data was applied in DL for the first time to achieve medical image analysis and AD prediction [36]. Recently, Armananzas *et al.* proposed the direct use of fMRI activation voxels of brain to tackle the automatic pattern analysis of AD and healthy individuals by applying different ML techniques to the classification of fMRI data for this purpose [37]. However, as far as we know, there is no literature to study this question from the view of functional connectivity of activity voxels.

Based on this consideration, in this paper, we propose a novel identification method based on the FCs between activity voxels in the whole brain, which is used to distinguish the AD patients from the health control (HC) groups. First, the classical ICA method is used to detect the activity voxels of the AD patients and HC subjects in the resting-state fMRI data. Second, the FCs between these activity voxels in the whole-brain are constructed, and then FCs with significant difference between AD and HC groups are identified by T-test with false discovery rate (FDR) correction, which are used as the input samples for the next classification. Finally, the machine learning techniques of SVM is adopted to classify the AD patients and HC subjects. The experimental results show that the proposed classification method in this paper can effectively distinguish AD patients and HC subjects, and can obtain higher classification accuracy.

The remainder of this paper is organized as follows: the proposed classification method will first be presented, followed by the experimental test which is designed to evaluate the performance of the classification method. Finally, the results and analysis will be presented together with interpretations and conclusions related to the advantages and limitations of this new classification method.

## II. Materials and Method

### A. Obtaining of Common Activated Voxels

Assuming that there are a total of $K$ subjects in the group, and all subjects have $T$ time points and $V$ voxels after normalization. In order to obtain the common activity voxels of subjects in the group. Firstly, we implement ICA on the single-subject level. For subject $i$, ICA is defined as:

\begin{equation*} { \boldsymbol {X}_{ \boldsymbol {i}}= \boldsymbol {M}}_{ \boldsymbol {i}} \boldsymbol {S}_{ \boldsymbol {i}},\quad \left ({i=1,2,\ldots,K }\right)\tag{1}\end{equation*}

where $\boldsymbol {X}_{ \boldsymbol {i}}$ represents the observed fMRI data, ${ \boldsymbol {S}_{ \boldsymbol {i}} =(\boldsymbol {s}_{ \boldsymbol {i1}}, \boldsymbol {s}_{ \boldsymbol {i2}},{\ldots,} \boldsymbol {s}_{ \boldsymbol {i} \boldsymbol {N}_{ \boldsymbol {i}}}{)}}^{T}$ is an $N_{i}\times V$ matrix, and each row represents an independent component (IC). Each column of $\boldsymbol {M}_{ \boldsymbol {i}} =(\boldsymbol {m}_{ \boldsymbol {i1}}, \boldsymbol {m}_{ \boldsymbol {i2}},{\ldots,} \boldsymbol {m}_{ \boldsymbol {i} \boldsymbol {N}_{ \boldsymbol {i}}} {)}$ which is a $T\times N_{i}$ mixing matrix represents the corresponding time courses.

Next, we calculate the location set of activity voxels in the ICs of each subject at a given threshold $\theta $, and denote it as ${ \boldsymbol {AVL}}_{ \boldsymbol {i}}$ for subject $i$. Then ${ \boldsymbol {AVL}}_{ \boldsymbol {i}}\left ({i=1,2,\ldots,K }\right)$ can be obtained as follows:

\begin{equation*} { \boldsymbol {AVL}}_{ \boldsymbol {i}}=\mathop {\bigcup }\nolimits _{j=1}^{N_{i}} \left \{{ v\vert {\begin{array}{l} abs\left ({\boldsymbol {s}_{ \boldsymbol {ij}}\left ({v }\right) }\right)\ge \theta, \\ v=1,2,\cdots,V;j=1,2,\ldots,N_{i} \\ \end{array}}}\right \}\tag{2}\end{equation*}

Finally, we can calculate the location set of common activity voxels in all subjects from ${ \boldsymbol {AVL}}_{ \boldsymbol {i}}\left ({i=1,2,\ldots,K }\right)$ and denote it as $\boldsymbol {CAVL}$, which can be obtained according to the following formula:

\begin{equation*} \boldsymbol {CAVL}=\mathop {\bigcap }\nolimits _{i=1}^{K} { \boldsymbol {AVL}}_{ \boldsymbol {i}}\tag{3}\end{equation*}

where $\boldsymbol {CAVL}$ is a $1\times N$ row vector and $N$ denotes the number of common activated voxels.

### B. Features Extraction for Classification

According to the method proposed in section “*A. Obtaining of common activity voxels*”, we firstly can obtain the location sets of common activity voxels of subjects in HC and AD groups, and denote them as ${ \boldsymbol {CAVL}}_{\mathbf {1}}$ and ${ \boldsymbol {CAVL}}_{\mathbf {2}}$ which include $N_{1}$ and $N_{2}$ voxels, respectively. Then the location set of common activity voxels of the two groups can be obtained, and denotes it as ${ \boldsymbol {CAVL}}_{\mathbf {12}}$ which include $N_{12}$ voxels.

\begin{equation*} { \boldsymbol {CAVL}}_{\mathbf {12}}={ \boldsymbol {CAVL}}_{\mathbf {1}}\bigcap { \boldsymbol {CAVL}}_{\mathbf {2}}\tag{4}\end{equation*}

Next, we calculate the FC between each pair of voxels in ${ \boldsymbol {CAVL}}_{\mathbf {12}}$ for each subject in each group, and denote the set of FCs as ${ \boldsymbol {FC}}_{ \boldsymbol {i}}$ for subject $i$ which is a $1\times {N_{12}\left ({N_{12}-1 }\right)} / 2$ row vector. In this study, FC is measured by the correlation coefficient, so that ${ \boldsymbol {FC}}_{ \boldsymbol {i}}$ can be calculated as follows:

\begin{align*} { \boldsymbol {FC}}_{ \boldsymbol {i}}=\left \{{{\begin{array}{l} corr\left ({\boldsymbol {X}_{ \boldsymbol {i}}\left ({{:, \boldsymbol {CAVL}}_{\mathbf {12}}\left ({p }\right) }\right), \boldsymbol {X}_{ \boldsymbol {i}}\left ({:,{ \boldsymbol {CAVL}}_{\mathbf {12}}\left ({q }\right) }\right) }\right) \\ p,q=1,\cdots,N_{12},p < q \\ \end{array}} }\right \} \\\tag{5}\end{align*}

where $corr\left ({\cdot }\right)$ represents the correlation operator, $\boldsymbol {X}_{ \boldsymbol {i}}$ is a $T\times V$ matrix represents the observed fMRI data for subject *i.*

Assuming that HC group and AD group include $K_{1}$ and $K_{2}$ subjects, respectively, and denotes the FCs of all subjects of HC group and AD group as $\boldsymbol {FC1}$ and $\boldsymbol {FC2}$.

Once $\boldsymbol {FC1}$ and $\boldsymbol {FC2}$ are obtained, the two-sample T-test is implemented on each FC to obtain the FCs with significant difference between AD group and HC group, and denotes the index set of FCs with significant difference as $\boldsymbol {DFCI}$ which can be obtained as follows:

\begin{equation*} \boldsymbol {DFCI}=\left \{{k\left \vert{ {\begin{array}{l} P_{ \boldsymbol {ttest}}\left ({\boldsymbol {FC1}\left ({:,k }\right) \boldsymbol {,FC2}\left ({:,k }\right) }\right){ < }P_{0},\\ k=1,2,\cdots,{N_{12}\left ({N_{12}-1 }\right)} \mathord {\left /{ {\vphantom {{N_{12}\left ({N_{12}-1 }\right)} 2}} }\right. } 2 \\ \end{array}}}\right.}\right \}\tag{6}\end{equation*}

where $P_{ \boldsymbol {ttest}}\left ({\cdot }\right)$ represents the P-value operator after FDR correction in T-test, $P_{0}$ represents the given level of significance which is 0.05 in our study. $\boldsymbol {FC1}\left ({:,k }\right)$ and $\boldsymbol {FC2}\left ({:,k }\right)$ represents the $kth$ column of $\boldsymbol {FC1}$ and $\boldsymbol {FC2}$, respectively.

**FIGURE 1.**
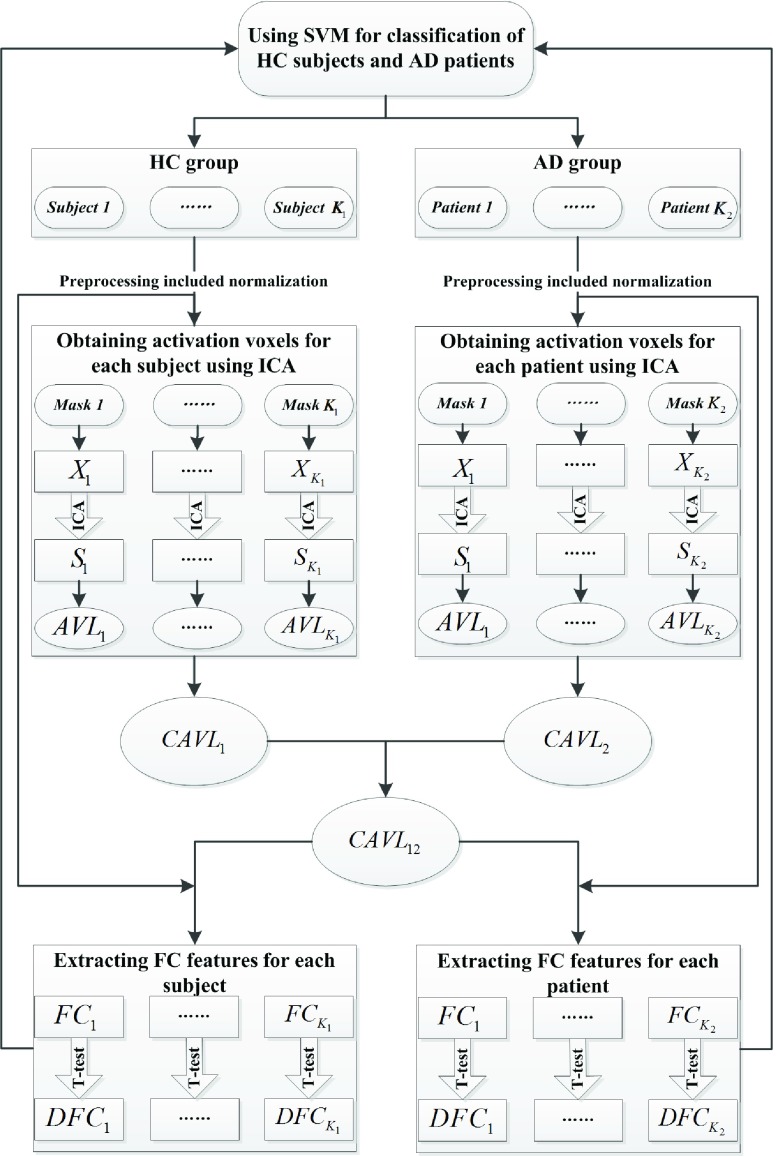
The chart flow of the proposed method. ${X}_{i}\left ({{i=1,2,\ldots,K_{j}}\vert {j=1,2}}\right) $ and ${S}_{i}\left ({{i=1,2,\ldots,K_{j}}\vert {j=1,2}}\right) $ represent the observed fMRI data within the brain mask and the ICs obtained using ICA for each subject respectively, and ${AVL}_{i}\left ({{i=1,2,\ldots,K_{j}}\vert {j=1,2}}\right) $ represents the location set of activity voxels for each subject. ${CAVL}_{\mathbf {1}}$ and ${CAVL}_{\mathbf {2}}$ represent the location set of common activity voxels for HC group and AD group respectively, and ${CAVL}_{\mathbf {12}}$ represents the location set of common activity voxels for HC and AD groups. ${FC}_{i}\left ({{i=1,2,\ldots,K_{j}}\vert {j=1,2}}\right) $ represents the FCs between the common activity voxels for each subject. ${DFC}_{i}\left ({{i=1,2,\ldots,K_{j}}\vert {j=1,2}}\right) $ represents the FCs with significant differences between HC and AD groups for each subject.

Finally, let $\boldsymbol {DFC1}$ and $\boldsymbol {DFC2}$ denotes the FCs with significant differences in HC group and AD group, which can be obtained according to the index set $\boldsymbol {DFCI}$ of FCs with significant difference between HC group and AD group. Once $\boldsymbol {DFC1}$ and $\boldsymbol {DFC2}$ are obtained, they are used as the input samples into the process of SVM for the classification of HC subjects and AD patients. The detailed schematic diagram of the implementation steps of the proposed method was showed in **Fig. 1**.

## III. Experiments and Data Processing

### A. Experimental Data

The resting-state fMRI datasets of sixty-seven patients with AD from 71 to 87 (mean = 77.30 ± 8.53) and seventy-six age-matched HC subjects from 71 to 87 (mean = 77.71 ± 5.71) from the Alzheimer’s Disease Neuroimaging Initiative (ADNI) database (*http://www.adni-info.org*) were used to evaluate the classification performance of the proposed method in this study. Data for this study were selected based on availability of resting-state fMRI datasets for age-matched HC subjects and patients with AD.

According to ADNI protocol (*http://adni.loni.usc.edu/*), the resting-state fMRI data was acquired on 3.0 Tesla Philips scanners while subjects were instructed to keep their eyes open. The images dataset was acquired using single-shot SENSE gradient an echo planar imaging (EPI) with 48 slices, providing whole-brain coverage and 140 volumes, a repetition time (TR) of 3s, an echo time (TE) of 30ms, a flip-angle (FA) of 80, and a scan resolution of $64\times 64$. The in-plane resolution was 3.31 mm $\times3.31$ mm, and the slice thickness was 3.31mm.

### B. Data Preprocessing

In this study, the preprocessing steps as well as all computations were running on the Matlab platform (Matlab, 2013a, Math-Works Inc., Sherborn, MA, USA). The fMRI data were preprocessed using the DPARSF software (*http://rfmri.org/DPARSF*), including removing the first 10 slices, slice timing, motion correction, spatial normalization and smoothing with the Gaussian kernel set to 4 mm, band-pass filtering (0.01-0.08Hz) and detrending. Meanwhile, the whole-brain signal was removed by a multiple linear regression analysis to reduce the effect of the physiological artifacts, and the head motion as well as the cerebrospinal fluid and white matter signals was removed as nuisance covariates to reduce the effects of motion and non-neuronal blood oxygen level-dependent fluctuations. Spatial ICA was implemented using FastICA algorithm [38] in all experiments. Particularly, ICASSO [39] with 20 runs of ICA and both ‘RandInit’ and ‘Bootstrap’ was used to obtain reliable ICs, and MDL [40] was used to estimate the number of ICs. Furthermore, the location and display of these networks were assessed using the MRIcro software (*http://www.mricro.com*).

## IV. Results and Analysis

In this study, we mainly consider the activity voxels in the gray matter region of brain. These activity voxels are obtained with threshold $\vert \text{z}\vert \ge2$ after z-scored the ICs of spatial ICA. **Table 1** shows the statistical distribution of activity voxels of AD and HC groups in the automated anatomical labeling (AAL) brain regions of cerebral cortex and their corresponding Montreal Neurological Institute (MNI) coordinates, which are considered as activity. Each brain region contains more than 10 voxels and accounts for more than 1% of the total voxels in the region.

**TABLE 1:** The Statistical Distribution of Activity Voxels of AD Group and HC Group in the AAL Brain Regions and Their Corresponding MNI Coordinates. The “Voxel Number” Represrnts the Number of Activity Voxels in the Brain Region, and the “Voxel Ratio” represents the Percentage of Obtained Activity Voxels Over the Total Voxels in the Corresponding Brain Region

AAL Regions	MNI coordinates (x, y, z)	AD group		HC group	
		voxel number	voxel ratio	voxel number	voxel ratio
Precentral_R	(41.37, −8.21, 52.09)	15	0.0150	–	–
Frontal_Sup_L	(−18.45, 34.81, 42.2)	109	0.1013	41	0.0381
Frontal_Sup_R	(21.9, 31.12, 43.82)	114	0.0984	34	0.0293
Frontal_Mid_L	(−33.43, 32.73, 35.46)	127	0.0877	75	0.0518
Frontal_Mid_R	(37.59, 33.06, 34.04)	126	0.0834	54	0.0358
Frontal_Mid_Orb_L	(−30.65, 50.43, −9.62)	–	–	17	0.0630
Frontal_Inf_Tri_L	(−45.58 29.91 13.99)	14	0.0193	–	–
Frontal_Inf_Orb_L	(−35.98, 30.71, −12.11)	12	0.0239	–	–
Frontal_Inf_Orb_R	(41.22, 32.23, −11.91)	17	0.0337	–	–
Supp_Motor_Area_L	(−5.32, 4.85, 61.38)	31	0.0473	–	–
Supp_Motor_Area_R	(8.62, 0.17, 61.85)	23	0.0345	–	–
Frontal_Sup_Medial_L	(−4.8, 49.17, 30.89)	91	0.1076	15	0.0177
Frontal_Sup_Medial_R	(9.1, 50.84, 30.22)	85	0.1326	13	0.0203
Frontal_Mid_Orb_L	(−5.17, 54.06, −7.4)	–	–	14	0.0622
Rectus_L	(−5.08 37.07 −18.14)	11	0.0421	10	0.0383
ParaHippocampal_L	(−7.63, −25.36, 70.07)	24	0.0839	–	–
ParaHippocampal_R	(25.38, −15.15, −20.47)	27	0.0854	–	–
Cuneus_L	(−5.93, −80.13, 27.22)	14	0.0312	23	0.0512
Lingual_R	(16.29, −66.93, −3.87)	19	0.0280	–	–
Postcentral_R	(41.43, −25.49, 52.55)	22	0.0193	–	–
Parietal_Sup_L	(−23.45, −59.56, 58.96)	–	–	18	0.0285
Parietal_Sup_R	(26.11, −59.18, 62.06)	52	0.0804	20	0.0309
Parietal_Inf_R	(46.46, −46.29, 49.54)	25	0.0597	–	–
Precuneus_L	(−42.46, −22.63, 48.92)	32	0.0297	39	0.0361
Precuneus_R	(9.98, −56.05, 43.77)	30	0.0321	26	0.0278
Temporal_Sup_R	(58.15, −21.78, 6.8)	14	0.0145	–	–
Temporal_Pole_Sup_L	(−39.88, 15.14, −20.18)	37	0.0969	40	0.1047
Temporal_Pole_Sup_R	(48.25, 14.75, −16.86)	70	0.1750	23	0.0575

It can be seen from **Table 1** that there are no activity voxels in the brain regions of Frontal_Mid_Orb_L (ORBmid.L/ORBsupmed.L) and Parietal_Sup_L in AD group compared with HC group. But there are also many brain regions that are individually activated in AD group such as Precentral_R, Frontal_Inf_Tri_L, Frontal_Inf_Orb (L/R), Supp_Motor_Area (L/R), ParaHippocampal (L/R), Lingual_R, Postcentral_R, Parietal_Inf_R and Temporal_Sup_R. At the same time, it can be found that the number of activity voxels in AD group is significantly increased in the brain regions of Frontal_Sup (L/R), Frontal_Mid (L/R), Frontal_Sup_Medial (L/R), Temporal_Pole_Sup_R compared with those in HC group. In addition, the common activity voxels of AD and HC groups are mainly located in the brain regions of Frontal_Sup (L/R), Frontal_Mid (L/R), Frontal_Sup_Medial (L/R), Cuneus_L, Parietal_Sup_R, Precuneus (L/R) and Temporal_Pole_Sup (L/R).

**FIGURE 2.**
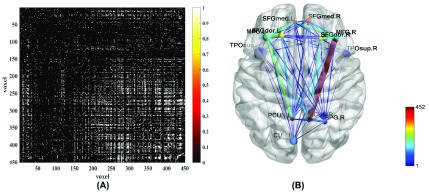
(A) the results of t-test on the FCs between the common activity voxels of AD group and HC group; (B) the spatial brain network between AAL regions corresponding to the activity voxels of FCs with significant differences.

**Fig. 2 (A)** shows statistical test results on the FCs between the common activity voxels of AD and HC groups, which are obtained by t-test with a confidence level of 95% after FDR correction. **Fig. 2 (B)** shows the spatial brain network between AAL regions corresponding to the activity voxels of FCs with significant differences, where the size of edge between different AAL regions represents the number of FCs with significant difference between the common activity voxels. The voxels number and ratio of these AAL regions and the corresponding MNI coordinates are shown in **Table 2**, which are more than 10 voxels and accounts for more than 1% of the total voxels.

**TABLE 2:** The Statistical Distribution of Activity Voxels With FCS of Significant Difference in the Aal Brain Regions and Their Corresponding MNI Coordinates. The “Voxel Number” Represrnts the Number of Activity Voxels in the Brain Region, and the “Voxel Ratio” Represents the Percentage of Obtained Activity Voxels Over the Total Voxels in the Corresponding Brain Region

AAL Regions	MNI coordinates	voxel number	voxel ratio
Frontal_Sup_L	(−18.45, 34.81, 42.2)	22	0.0204
Frontal_Sup_R	(21.9, 31.12, 43.82)	17	0.0147
Frontal_Mid_L	(−33.43, 32.73, 35.46)	42	0.0290
Frontal_Mid_R	(37.59, 33.06, 34.04)	32	0.0212
Frontal_Sup_Medial_L	(−4.8, 49.17, 30.89)	10	0.0118
Frontal_Sup_Medial_R	(9.1, 50.84, 30.22)	11	0.0172
Cuneus_L	(−5.93, −80.13, 27.22)	13	0.0290
Parietal_Sup_R	(26.11, −59.18, 62.06)	11	0.0170
Precuneus_L	(−42.46, −22.63, 48.92)	14	0.0130
Precuneus_R	(9.98, −56.05, 43.77)	19	0.0203
Temporal_Pole_Sup_L	(−39.88, 15.14, −20.18)	20	0.0524
Temporal_Pole_Sup_R	(48.25, 14.75, −16.86)	11	0.0275

It can be seen clearly from **Fig. 2 (B)** that the number of FCs between Frontal_Sup_L and Frontal_Mid_L, Frontal_Sup_R/Frontal_Sup_Medial_R and Frontal_Mid_R, Frontal_Mid (L/R) and Frontal_Sup_Medial_L, Frontal_Sup_L/Frontal_Mid (L/R) and Precuneus_L, Frontal_Sup_R/Frontal_Mid (L/R)/Parietal_Sup_R and Precuneus_R are higher than those between other regions. At the same time, there is almost no FCs between Frontal_Sup_L/Frontal_Sup_Medial_R/Precuneus (L/R) and Cuneus_L, Frontal_Sup_R/Frontal_Sup_Medial (L/R)/Cuneus_ L/Precuneus (L/R)/Temporal_Pole_Sup_R and Temporal_Pole_Sup_L, Frontal_Sup_R/Parietal_Sup_R/Cuneus_L/Frontal_Sup_Medial_R/Precuneus (L/R) and Temporal_Pole_ Sup_R.

**FIGURE 3.**
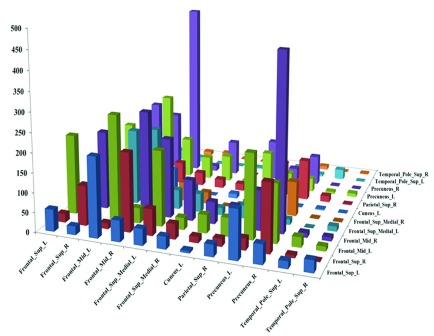
The number of FCs with significant differences between the AAL regions corresponding to the activity voxels of FCs with significant differences.

These results can also be found from **Fig. 3**, which shows the number of FCs with significant differences among the common activity voxels between different AAL regions in **Table 2**. We can draw the similar conclusions of **Fig. 2** from **Fig. 3**. Moreover, it can be further found from the figure that there are many FCs with significant differences between the common activity voxels of AD group and HC group in each AAL brain region except for the regions of Cuneus_L, Parietal_Sup_R and Temporal_Pole_Sup_R.

**FIGURE 4.**
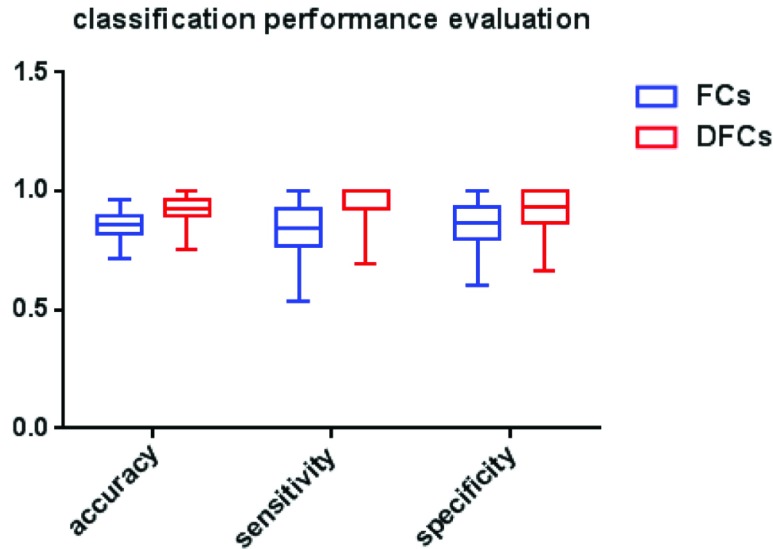
The classification accuracy, sensitivity and specificity using all FCs and FCs with significant differences (DFCs) between the common activated voxels of AD group and HC group.

**Fig. 4** shows the classification accuracy, sensitivity and specificity of AD and HC subjects, which obtained using SVM on all FCs and FCs with significant differences between the common activity voxels of AD and HC groups. In particular, the value of parameter C = 1 and linear kernel function were used in the SVM, and the cross-validation was used to control the over-fitting problem [41]. Specifically, the dataset was randomly divided into halves, 80% of which were used as training and the rest 20% were used as testing, and this process was run 1000 times. The average accuracy of the 1000 times was used as the final results, and the sensitivity and specificity of these 1000 results were also given simultaneously, as shown in the figure. It can be seen clearly from the figure that the classification accuracy, sensitivity and specificity obtained using FCs with significant differences are significant higher than those obtained using all FCs between the common activity voxels of AD and HC groups, which means that the FCs with significant differences between the common activity voxels play an important role in the classification of AD and HC subjects and has a potential value for the AD neural pathogenesis research.

**FIGURE 5.**
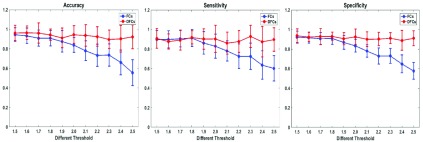
The classification accuracy, sensitivity and specificity using all FCs and FCs with significant differences (DFCs) between the common activity voxels on the cases of different threshold parameters, which ranges from 1.5 to 2.5 with a step-length of 0.1.

In order to evaluate whether the change of this parameter affects the stability of SVM classification results, multiple thresholds are adopted for the study in this paper, and the classification results of SVM are shown in **Fig. 5**. It can be seen from the figure that the change of threshold parameters does not affect the robustness of SVM classification results, and the advantages of classification performance become more and more obvious with the increase of threshold parameters, especially for the cases of threshold parameter larger than 2.0. Meanwhile, in order to verify the classification effect of SVM, we compared the classification results of Linear SVM (LSVM) with a variety of classification algorithms in the revised manuscript according to the comment of reviewer, including Decision Tree (DT), Linear Discriminant (LD), Logistic Regression (LR), K-Nearest Neighbor (KNN), and Gaussian SVM (GSVM), as shown in **Table 3**. It can be seen clearly that the classification effect of LSVM is better than that of other methods regard to accuracy, sensitivity and specificity.

**TABLE 3:** The Classification Performance of Multiple Algorithms, Including DT, LD, LR, KNN, GSVM, and LSVM

	accuracy	sensitivity	specificity
DT	57.10%	46.15%	66.67%
LD	82.10%	76.92%	86.67%
LR	78.60%	84.62%	73.33%
KNN	78.60%	100%	60%
LSVM	92.90%	100%	86.67%
GSVM	89.30%	92.31%	86.67%

**FIGURE 6.**
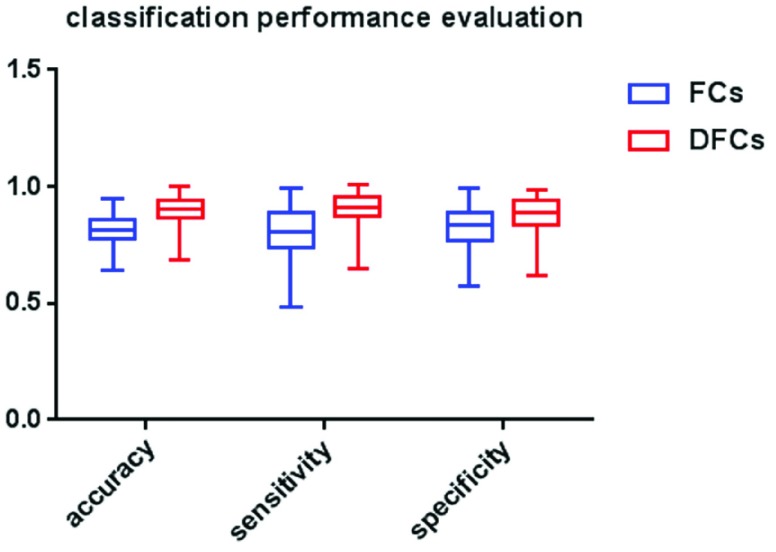
The classification accuracy, sensitivity and specificity using all FCs and DFCs between the common activated voxels of MCI and HC groups.

## V. Discussion

Currently, the most studies are mainly focus on the activation degree of brain regions, brain networks or voxels as well as the FC differences between them in the fMRI data analysis of AD, but there are few studies to explore the difference between AD and HC groups from the perspective of activity voxel. Recently, Armananzas *et al.* used the activity voxels as features to classify AD patients from healthy subjects, and achieved a good recognition effect [37]. However, they did not explore how FCs between these activity voxels differs in healthy subjects and AD patients. Moreover, we can easily obtain the differences for different activity voxels between AD and HC groups, but whether the common activity voxels show the consistency between them, there are no relevant researches at present. In addition, Irajia *et al.* showed that the analysis of fMRI data in the connectivity domain had more advantageous than that in the time domain, and could better explain the correlation rules of neurological diseases such AD [42].

Therefore, the main contribution of this study is to explore the distribution of activity voxels and FCs between them in AD and HC groups, and to analyze the FCs of common activity voxels between AD and HC groups in the connectivity domain, and to study their roles in distinguishing between AD patients and healthy subjects, so as to provide a certain reference basis for exploring the neuropathogenesis of AD. First, we made a statistical analysis of the distribution of activated voxels for the two groups of subjects in the AAL template, as shown in **Table 1**, in which the activity voxels were obtained by the method proposed in this paper, and elaborated on the cognitive function of the brain areas related to these activity voxels and the correlation with AD research.

Although the information about brain activity in AD patients can be obtained directly by comparing the differences of brain regions or activity voxels between AD and HC groups, there is no relevant conclusion on whether the common activity voxels are consistent in AD patients and healthy subjects. Moreover, recent studies have shown that the analysis of fMRI data in the connectivity domain has more advantageous than that in the time domain, which can be better used to explain the related laws of neurological diseases such as AD [42]. Therefore, we also conducted a detailed and in-depth analysis on the FCs between the same activity voxels of the two groups of subjects in addition to directly analyze the distribution of activated voxel in the AD and HC groups as well as the related neurocognitive functions, and found the FCs with significant differences between AD and HC groups.

Then, these FCs with significant differences and the distribution of related activity voxels in the AAL brain regions were analyzed, which were consistent with previous studies about AD. In order to further verify the dissimilarity of the FCs with significant difference between AD patients and HC subjects and the significance of them in studying neuromechanism of AD, they were used as features to classify AD patients and HC subjects through SVM. SVM is a supervised machine learning algorithm derived from binary classification problem. Its advantage is that it can get much better results than other algorithms on a small sample training set, with strong generalization ability, fast classification speed and easy interpretation of results. This is because its optimizations object aims to minimize structural risk, not empirical risk, thus reducing the requirement for data size and data distribution. The results showed that these FCs have a good discrimination performance in recognition of AD patients from HC subjects compared with all FCs between the common activity voxels of the two groups, thus providing certain reference significances for the AD research at voxel level.

Many methods have been used for the study of AD classification, such as Deep Learning, AdaBoost, Support Vector Machines and Convolutional Neural Network [33]–[36], which have achieved a good classification effect, but they used different classification features, so there were different problems. For example, it is easy to ignore the boundary information between structural regions and functional regions when using 90 brain regions in AAL template to construct classification features from the fMRI data of AD, and also easy to miss the functional integration information of other brain regions on AD when specific brain functional regions are used. In addition, although recent studies have explored AD from the perspective of activated voxels [37], they have not considered the role of FCs between these activated voxels in AD prediction, and studies have shown that fMRI data analysis in the connectivity domain had more advantageous than that in time domain [42]. Therefore, this paper carried out relevant studies on AD based on the FCs between activated voxels, and achieved a good classification accuracy, which has a certain significance for the clinical diagnosis of AD.

As a classical method for detecting brain functional connectivity, ICA is a blind source separation method based on higher order statistical moments. The purpose of ICA is to decompose the observed multivariate data into the source signals which are assumed statistically independent and non-Gaussian, and it has been successfully applied to study of fMRI data and to find the underlying independent sources. Therefore, it was adopted to detect the activity voxels in this study, in which the number of ICs was estimated by MDL. When using ICA to obtain the activity voxels for each subject, the brain mask was built automatically from the data itself, and then the fMRI data within the mask was used to obtain the activity voxels. Since all subjects had the same amount of voxels after the preprocessing of spatial normalization, so that they were corresponding to the same spatial locations in the standardized space. Once the activity voxels of each subject and their corresponding positions in the standardized space are obtained, we can calculate the common activity voxels of all subjects to represent the common active areas of the corresponding group. In fact, although there are some structural differences in the brains of each subject, most areas remain the same in the standardized space, so this method allows us to preserve some individual differences while also maximizing their commonality at the activity voxel level.

In the process of obtaining activity voxels, the activity voxels in the brain region for each subject were used to preserve the individual specificity, while the common activity voxels of all subjects were used as the common activity region on behalf of the corresponding subjects in the group, such as AD group and HC group in our study. Furthermore, the common activity voxels need to be further screened through the known structural templates such as the AAL template, to determine which activity voxel is really caused by brain information. Only those voxels that belong to corresponding brain regions and reach a certain amount and proportion can be selected as the truly meaningful activity voxels, as shown in **Table 1** of this paper. It can be seen from the table that these voxels are located in areas of the brain associated with certain cognitive functions.

This may be because the noise-induced activity voxel is less likely to occur in all ICs at once than in real neural signals. In general, the activity voxels with physiologically significant have little difference at the results of different running times, while the noise-induced pseudoactivator voxels vary greatly between subjects. Currently, there have been a number of preliminary studies in this area. For example, Armananzas *et al.* proposed the direct use of fMRI activation voxels of brain to tackle the automatic pattern analysis of AD and healthy individuals by applying different machine learning techniques to the classification of fMRI data for this purpose [37]. Therefore, the activity voxel detection method proposed in this paper can retain the useful voxels of physiological significance to the maximum extent and remove the voxels caused by noise. These voxels not only depict the differences between individuals, but also reflect the common characteristics at the group level.

In addition, it is worth noting that the classification method proposed in this paper may be related to some factors, such as the number of subjects in AD and HC groups and the number of ICs used in ICA. Different activity voxels may be obtained due to the different number of subjects or ICs, and it further leads to different FCs samples. Meanwhile, it needs to predefine a threshold parameter $\theta $ in formula (2) as the standard to determine whether a voxel is activity, and different voxels may be obtained with different threshold $\theta $ in the process of activity voxel calculation. In this study, $\theta =2$ was selected as the threshold parameter to determine the activity voxels in the ICs after z-score transformation.

In order to illustrate the effectiveness of FCs samples selection in the proposed classification method, the FCs between the common activity voxels of AD and HC groups are used as the input samples for SVM classification. However, compared with the using of FCs with significant differences as input samples, the classification accuracy is significantly lower. Furthermore, the sample size of FCs with significant differences is far lower than that of FCs between the common activity voxels, which reduces the computational complexity of SVM in classification. In other words, the original sample contains many FCs that have no practical effect on classification. Therefore, the proposed classification method is expected to provide a new way for the clinical auxiliary diagnosis and prediction of AD.

In this paper, the fMRI data of AD and HC groups in this study were downloaded from public database ADNI (http://adni.loni.usc.edu/), which had the same fMRI data scanning parameters and similar demographic information. But there was no fMRI data of mild cognitive impairment (MCI) with the corresponding conditions, so only the fMRI data of AD and HC were used for this study, which was indeed a deficiency. Therefore, in order to illustrate the significance of the proposed method in the diagnosis of MCI, we used the fMRI data of 50 patients with MCI from ADNI database (http://www.adni-info.org) to evaluate the performance of the proposed method, and the data description and results analysis were presented in APPENDIX.

## VI. Conclusion

In this paper, we proposed a new method for the identification of AD patients from HC subjects by using the FCs between the activity voxels in the fMRI data. ICA was firstly used to detect the common activity voxels from the fMRI data of AD and HC groups, and then the FCs of subjects in the two groups between these voxels were obtained. Finally, two sample T-test was used to find the FCs with significant differences between AD group and HC group, and then those were further used as the feature samples of SVM for the classification of AD patients. The experimental results demonstrated that the proposed method could obtain a high classification accuracy for AD patients, and also showed that more regions and more voxels in some brain regions were activity in the brain of AD patients, which may be due to the reason of compensation mechanism in the brain, and it provided a new perspective for the study of AD pathogenesis.

According to the statistical distribution of activity voxels of AD and HC groups in **Table 1**, the results showed that more brain regions of Inferior frontal gyrus, Supplementary motor area, Parahippocampal gyrus, Lingual gyrus, Postcentral gyrus, Inferior parietal, Precuneus and Superior temporal gyrus were active in AD group compared with HC group, although there were no activity voxels in Frontal_Mid_Orb_L of Middle frontal gyrus, Frontal_Mid_Orb_L of Superior frontal gyrus, and Parietal_Sup_L of Superior parietal gyrus. They were mainly located in Prefrontal lobe, Parietal lobe, Temporal lobe, Occipital lobe and other parts of Frontal lobe. In addition, more voxels in Superior frontal gyrus, Middle frontal gyrus, Superior frontal gyrus and Superior temporal gyrus (Temporal pole) were activity in AD group, and they were mainly in Prefrontal lobe and Temporal lobe. These results were consistent with previous studies about AD, which reports significant changes in these brain regions, and the changes of FCs within each brain lobe as well as those between brain lobes in AD [43]–[46].

Moreover, the results in **Fig. 4**–**Fig. 5** showed that the identification performance using only FCs with significant difference between the activity voxels was better than that using all FCs between the activity voxels in the classification of AD patients and HC subjects. According to the distribution of the activity voxels related to FCs with significant difference shown in **Table 2**, they were mainly located in the brain regions of Superior frontal gyrus, Middle frontal gyrus, Superior frontal gyrus, Superior parietal gyrus, and Precuneus. Based on the statistics of the number of FCs with significant difference between different brain regions in **Fig. 3**, we found that the FCs between activity voxels within prefrontal lobe as well as those between prefrontal and parietal lobes played an important role in the classification prediction of AD patients, which were shown in **Fig. 2**. Therefore, we will further use more AD, MCI and HC fMRI data to evaluate the effectiveness of the proposed algorithm in the future, which may be providing a new perspective for the study of AD pathogenesis mechanism.

## Appendix

The resting-state fMRI datasets of 50 patients with MCI from 66 to 78 (mean = 72.40± 6.32) from ADNI database were used to evaluate the classification performance of the proposed method in this study. According to ADNI protocol, the resting-state fMRI data was acquired on 3.0 Tesla Philips scanners. The images dataset was acquired using single-shot SENSE gradient an EPI with 38 slices, providing whole-brain coverage and 160 volumes, a TR of 3s, a TE of 30ms, a FA of 90, and a scan resolution of $64\times 64$. The in-plane resolution was 3.39 mm $\times3.39$ mm, and the slice thickness was 3.39 mm.

After these MCI data have processed by the same data preprocessing steps in Section B “Data preprocessing” of III “EXPERIMENTS AND DATA PROCESSING”, the FCs and DFCs features corresponding to MCI and NC groups are obtained by using the method proposed in this paper. Then, we also used SVM with linear kernel function to classify MCI patients and HC subjects, and the classification results were shown in **Fig.s1**. **Fig.s1** showed the classification accuracy, sensitivity and specificity of MCI and HC subjects, which obtained using SVM on all FCs and DFCs between the common activity voxels of MCI and HC groups. It can be seen clearly from the figure that a better classification accuracy, sensitivity and specificity has been obtained by using DFCs between the common activity voxels of MCI and HC groups, which means that the DFCs between the common activity voxels play an important role in the classification of MCI and HC subjects and has a potential value for the MCI diagnosed.
